# Keratinocyte Growth Factor 2 Ameliorates UVB-Induced Skin Damage *via* Activating the AhR/Nrf2 Signaling Pathway

**DOI:** 10.3389/fphar.2021.655281

**Published:** 2021-06-07

**Authors:** Shuang Gao, Keke Guo, Yu Chen, Jungang Zhao, Rongrong Jing, Lusheng Wang, Xuenan Li, Zhenlin Hu, Nuo Xu, Xiaokun Li

**Affiliations:** ^1^ School of Pharmaceutical Sciences, Wenzhou Medical University, Wenzhou, China; ^2^ College of Life and Environmental Sciences, Wenzhou University, Wenzhou, China

**Keywords:** keratinocyte growth factor 2, ultraviolet B, photoprotective, aryl hydrocarbon receptor, Nrf2

## Abstract

**Objective:** Exposure to ultraviolet B (UVB) can cause skin damage through oxidative stress, DNA damage, and apoptosis. Keratinocyte growth factor (KGF) has been shown to reduce the content of intracellular reactive oxygen species (ROS) following UVB exposure, a role that is crucial for the efficient photoprotection of skin. The present study evaluated the photoprotective effect of KGF-2 on UVB-induced skin damage and explored its potential molecular mechanism.

**Methods:** To evaluate the effect of KGF-2 on UVB-induced damage *ex vivo*, a human epidermal full-thickness skin equivalent was pretreated without or with KGF-2 and then exposed to UVB and the levels of histopathological changes, DNA damage, inflammation, and apoptosis were then evaluated. The ability of KGF-2 to protect the cells against UVB-inflicted damage and its effect on ROS production, apoptosis, and mitochondrial dysfunction were determined in HaCaT cells.

**Results:** Pretreatment of the epidermis with KGF-2 ameliorated the extent of photodamage. At the cellular level, KGF-2 could attenuate ROS production, apoptosis, DNA damage, and mitochondrial dysfunction caused by UVB exposure. KGF-2 could also activate the aryl hydrocarbon receptor (AhR) to trigger the Nrf2 signaling pathway.

**Conclusion:** Taken together, our findings suggested that KGF-2 could ameliorate UVB-induced skin damage through inhibiting apoptosis, reducing oxidative stress, and preventing DNA damage and mitochondrial dysfunction *via* regulating AhR/Nrf2 signaling pathway.

## Introduction

The skin, the largest organ of the human body, is divided into three different layers, called subcutis, dermis, and epidermis. The outermost epidermis is mainly composed of keratinocytes and it provides a selective physical barrier to protect human beings from harmful substances that come from the environment (Eckhart and Zeeuwen 2018). Ultraviolet (UV) radiation is the most ubiquitous environmental threat that affects human skin (Perluigi et al., 2010). UV is composed of three different types classified by wavelengths: UVA (320–400 nm), UVB (280–320 nm), and UVC (200–280 nm). UVC is effectively blocked from reaching the Earth’s surface by the ozone layer of the atmosphere. UVA can penetrate deeply into the dermis to cause aging and wrinkling of the skin. UVB on the other hand is regarded as “burning rays” and it is almost completely absorbed by the epidermis, where it can induce oxidative damage, inflammation, DNA damage, photoaging, and apoptosis (Tomaino et al., 2006; Oh et al., 2016; D'Orazio et al., 2013; de Gruijl 2000).

The hallmark of UVB-induced skin damage is the formation of apoptotic keratinocytes within the epidermis once the dose of UV exceeds a certain threshold (Takahashi et al., 1997). Such apoptotic keratinocytes, which can be identified by their pyknotic nuclei and cytoplasmic shrinkage (Bayerl et al., 1995; D'Orazio et al., 2013). UVB-induced DNA damage and the formation of reactive oxygen species (ROS) are thought to be crucial factors that trigger the apoptotic machinery (Wang et al., 1999). Direct UVB-induced DNA damage in keratinocytes consists of two major forms of lesions: cyclobutane pyrimidine dimers (CPDs) and 6–4 pyrimidine photoproducts (6–4 PPs) (Garinis et al., 2005; Rastogi et al., 2010). Such DNA damage can also induce an inflammatory response through mediating the release of interleukin 1β (IL-1β), IL-6 and tumor necrosis factor α (TNF-α) (Piotrowska et al., 2016; Prasad and Katiyar 2017). UVB can promote the excessive production of ROS in keratinocytes, and the increased level of ROS can in turn, inhibit the antioxidant defense mechanism as well as stimulate the inflammatory response in skin (Darr and Fridovich, 1994; Kuanpradit et al., 2017). Meanwhile, ROS can attack the mitochondrial membranes and mitochondrial DNA directly, causing mitochondrial dysfunctions (Kudryavtseva et al., 2016).

Keratinocyte growth factor (KGF) is a fibroblast growth factor (FGF) family member and it is known to protect skin against oxidant injury (Werner et al., 2007). KGF-1 plays a crucial protective role in keratinocytes through reducing the level of intracellular ROS and apoptosis following UVB exposure (Braun et al., 2006; Kovacs et al., 2009). A previous study has reported that exposure to UVB and oxidant stimuli can trigger the activation and internalization of KGFR, similar to those induced by KGF-1 (Marchese et al., 2003). Fibroblast growth factor-10 (KGF-2), another member of the FGF family, is similar to KGF-1 in structure and function. KGF-2 promotes both the growth and differentiation of keratinocyte cells (Soler et al., 1999). KGF-2 can suppress excessive oxidative stress-induced cell apoptosis to promote cell/tissue regeneration (Dong et al., 2019). Additionally, KGF-2 can augment the repair of oxidant-induced DNA damage in alveolar epithelial cells (Takeoka et al., 1997; Upadhyay et al., 2004). We, therefore, hypothesized that KGF-2 would protect the skin against UVB-induced damage following its exposure. In this study, the involvement of KGF-2 in UVB-induced skin damage was evaluated and the underlying mechanism was explored.

## Materials and Methods

### Reagents

KGF-2 was expressed in *Escherichia coli* and purified in our laboratory*.* GNF 351 was purchased from Selleck (Shanghai, China).

### Detection of Human Epidermal Equivalents After Ultraviolet B Irradiation

Human epidermal equivalent (HEE) is an *in vitro* reconstructed human epidermis from normal human keratinocytes cultured on an inert polycarbonate filter at the air-liquid interface, in a chemically defined medium. This model exists at different stages of maturity. This model is histologically similar to *in vivo* human epidermis. HEE (Biocell Biotechnology, Guangdong, China) was placed in a six-well cultured plate and equilibrated in HEE medium for 24 h at 37°C in a 5% CO_2_ incubator before use. The HEE was treated with KGF-2 for 4 h *via* topical application. The medium was then replaced with PBS, the HEE was then exposed to 200 mJ/cm^2^ of UVB radiation. After exposure, it was cultured in fresh medium for 36 h and then harvested. The HEE was fixed in 4% paraformaldehyde, dehydrated, and then embedded in paraffin, followed by sectioning into 5 μm-thick coronal slices. The slices were then subjected to the following analyses.

### Histological Examination

For histological examination, the HEE slices were stained with hematoxylin-eosin and then examined by fluorescence microscopy using a DM3000 microscope (Leica, Wetzlar, Germany) to assess the histological changes. The number of epidermal UVB-damaged keratinocyte characterized by the presence of pyknotic condensed nuclei in the HEE sample was quantified.

### Immunofluorescence Assay

For immunofluorescence assay, the HEE slices were dewaxed, rehydrated, and incubated in 3% H_2_O_2_/methanol solution for 25 min and then blocked with 5% BSA for 4 h at 37°C. After that, the slices were incubated with the immunofluorescence markers: phospho-H2AX (1:1,000) (Cell Signaling Technology, Beverly, MA, United States) and anti-CPD (1:200) (Cosmo Bio, Tokyo, Japan) overnight at 4°C in a humidified chamber. The samples were then washed three times with PBS, followed by incubation with Alexa Flour 488 (green) donkey anti-rabbit secondary antibody and Alexa Flour 568 (red) donkey anti-mouse secondary antibody for 1 h at room temperature in the dark. Finally, the samples were stained with ProLong Gold Antifade reagent containing DAPI (Life Technologies Corporation, NY, United States) and then mounted on glass slides and examined under a Ti2-E&CSU-W1 confocal microscope (Nikon, Tokyo, Japan). The 488 nm laser intensity is 2% with the detector voltage of 430 V. The 561 nm laser intensity is 4% with the detector voltage of 550 V.

### TUNEL Assay

TUNEL assay carried out for the HEE slices according to the instructions of manufacturer (Roche, Penzberg, Germany) and examined under a Ti2-E&CSU-W1 confocal microscope (Nikon, Tokyo, Japan). The 488 nm laser intensity is 5% with the detector voltage of 500 V.

### Cells Culture

Immortalized human keratinocytes were supplied by Zhong Qiao Xin Zhou Biotechnology Co., Ltd. (Shanghai, China). The cells were cultured in Dulbecco’s modified Eagle’s medium (DMEM) (GIBCO, Life Technologies Corporation, NY, United States) containing 10% fetal bovine serum (FBS) (GIBCO, Life Technologies Corporation, NY, United States) at 37°C in a 5% CO_2_ incubator.

### Ultraviolet B Irradiation

HaCaT cells were incubated with KGF-2 at different concentrations (0, 50, 200 ng/ml) for 4 h, and then washed with PBS. The cells were then exposed to UVB (200 mJ/cm^2^) in PBS using a VL6-M Biotronic device (Vilber Lourmat, Marne La Vallee, France). After UVB exposure, the cells were incubated with fresh DMEM at 37°C in a 5% CO_2_ incubator for different times depending on the experiment*.*

### Intracellular Reactive Oxygen Species Measurement

HaCaT cells were treated under the designated experimental conditions. An assay kit (Beyotime, China) was employed to measure ROS production according to the manufacturer’s protocol. Briefly, after 1 h of UVB (200 mJ/cm^2^) irradiation, the cells were washed twice with PBS, and then incubated with DCFH-DA at a final concentration of 5 μM for 20 min at 37°Cin a 5% CO_2_ incubator. After that, the cells were digested and washed three times with PBS, and intracellular ROS production was then measured with an ACEA NovoCyte flow cytometer (Agilent, Santa Clara, CA, United States), and the result was analyzed using NovoExpress software.

### Apoptosis Assay

HaCaT cells were cultured in a 6-well plates at a density of 5 × 10^4^ cells/well for 24 h and then incubated with KGF-2 at different concentrations (0, 50, 200 ng/ml) for 4 h followed by exposure to UVB (200 mJ/cm^2^). After that, the cells were incubated with fresh DMEM at 37°C in a 5% CO_2_ incubator for 6 h. An annexin V &FITC apoptosis detection kit (Dojindo Laboratories, Kumamoto, Japan) was used to stain the cells according to the manufacturer’s protocol. The stained cells were quantified with an ACEA NovoCyte flow cytometer (Agilent, Santa Clara, CA, United States). The apoptotic frequency was expressed as the percentage of Annexin V-FITC positive cells.

### JC-1 Staining

JC-1 fluorescent probes (Beyotime, Jiangsu, China) was used to determine the mitochondrial membrane potential (MMP). First, HaCaT cells were cultured for 24 h in 6-well plates at a density of 5 × 10^4^/well. Next, the cells were treated with the different concentrations of KGF-2 (0, 50, 200 ng/ml) for 4 h and then irradiated with UVB followed by 1 h of incubation. After that, the cells were washed with PBS and incubated with DMEM containing 5 μM of JC-1 for 20 min. Finally, the cell nuclei were stained with 40,6-diamidino-2-phenylindole (DAPI), and the MMP of the cells was monitored with a fluorescence microscope (Nikon, Tokyo, Japan) (Red: excitation/emission 530 nm/590 nm, exposure time: 180 ms; Green: excitation/emission 490/530 nm, exposure time: 200 ms).

### Mitochondrial Mass Analysis

HaCaT cells were treated in the same way as described for JC-1 staining. The fluorescent probe Mito-Tracker Green (Beyotime, Jiangsu, China) was used to determine the mitochondrial mass of HaCaT cells. In brief, the cells were first incubated with 50 nm Mito-Tracker Green in DMEM for 1 h at 37°C in the dark. After that, they were washed with PBS, and the nuclei were stained with DAPI, while the fluorescence of the mitochondria was measured with a Ti2-E&CSU-W1 confocal microscope (Nikon, Tokyo, Japan) using λEm and λEx of 488 and 590 nm, respectively. The 488 nm laser intensity is 3% with the detector voltage of 450 V.

### Quantitative Real-Time PCR

HEE was treated without or with KGF-2 followed by UVB irradiation as described above and total RNA was then isolated from the sample using TRIzol reagent (Invitrogen, Carlsbad, CA, United States) according to the manufacturer’s instructions. At the same time, total RNA was also isolated from HaCaT cells treated without or with KGF-2 and followed by UVB irradiation. The concentration of total RNA obtained was determined with a NanoQuant Plate (Tecan, Mannedorf, Switzerland). Reverse transcription was performed with PrimeScript RT reagent Kit (Takara, Dalian, China) using 1 μg of total RNA as template. *q*RT-PCR was performed using the SYBR Green Master Mix (Applied Biosystems, Foster City, CA) and the LC96 system (Roche, Basel, Switzerland). The sequences of all the primers used are shown in Table 1. The expression of target genes was normalized to the level of β-actin, which served as an endogenous control. The relative expression of each targeted gene was analyzed using the 2^−ΔΔCt^ method.

**TABLE 1:** Primer sequences used in this study.

Gene name	Primer sequences
β-ACTIN	F: 5′- CTC CAT CCT GGC CTC GCT GT -3′
	R: 5′- GCT GTC ACC TTC ACC GTT -3′
TNF-α	F: 5′-CCT GTA GCC CAC GTC GTA GC-3′
	R: 5′-TTG ACC TCA GCG CTG AGT TG-3′
IL-1β	F: 5′-TGG CAA TGA GGA TGA CTT GT-3′
	R: 5′-GTG GTG GTC GGA GAT TCG TA-3′
IL-6	F: 5′-CCG AGA AGG AGA CTT CAC AG-3′
	R: 5′-TCC ACG ATT TCC CAG AGA AC-3′

### Immunofluorescence Staining for Detection of γ-H2AX and CPD

HaCaT cells grown on coverslips (WHB Scientific, Shanghai, China) for 24 h and treated with KGF-2 or exposed to UVB as described above and allowed to recover for 1 h at 37°C in a 5% CO_2_ incubator. The cells were then washed three times with PBS and immediately fixed in 4% paraformaldehyde for 15 min at room temperature. The fixed cells were permeabilized with 0.5% Triton X-100 in PBS and immersed in 2 M HCl for 30 min at room temperature. After being washed with TBS, the cells were blocked with 5% BSA in TBST (TBS + 0.1% Triton X-100) for 30 min at 37°C with gentle shaking. For the double staining experiments, the cells were incubated with γ-H2AX and anti-CPD in 5% BSA for overnight at 4°C. After that, the cells were washed three times with TBST, incubated with a secondary antibody, and then mounted with an antifade reagent containing DAPI. The fluorescence of the cells was measured using a Ti2-E&CSU-W1 confocal microscope (Nikon, Tokyo, Japan). The 488 nm laser intensity is 1% with the detector voltage of 450 V. The 561 nm laser intensity is 3% with the detector voltage of 500 V.

### Western Blot

HaCaT cells were cultured in a 6-well plates at a density of 5 × 10^4^/well for 24 h and then treated with different concentrations of KGF-2 (0, 50, and 200 ng/ml) for 4 h followed by exposure to UVB. After UVB irradiation, the cells were incubated at 37°C in a 5% CO_2_ incubator for 30 min. Cytoplasmic and nuclear proteins were extracted from the cells using NE-PER™ Reagents (Thermo scientific, NY, United States) according to the manufacturer’s instructions. The samples were then subjected to SDS-PAGE and the protein bands in the gel were transferred to a PVDF membrane. The membrane was immunolabeled with one of the following primary antibodies: anti-Nrf2, anti-AHR, anti-capase-3, anti-capase-9, anti-CYP1A1, anti-LaminB1, or anti-Tubulin (Cell Signaling Technology, Beverly, MA, United States) followed by washing and further incubation with a secondary antibody (Cell Signaling Technology, Beverly, MA). The immune complexes were finally detected with a chemiluminescence substrate (Thermo scientific, NY, United States) and the image was captured with an Amersham Imager (GE Healthcare Biosciences, Pittsburgh, PA, United States).

### Statistical Analysis

All quantitative data were presented as means ± SD from at least three independent experiments. A comparison of multi-group data was carried out with a One-way analysis of variance (ANOVA) followed by Dunnett’s test. Statistical significance was considered at either the *p* < 0.05 or *p* < 0.01 level. All graphical presentations were done using GraphPad Prism 5.01 (GraphPad, San Diego, CA, United States).

## Results

### Keratinocyte Growth Factor-2 Ameliorated the Extent of Photodamage of Epidermis

**FIGURE 1:**
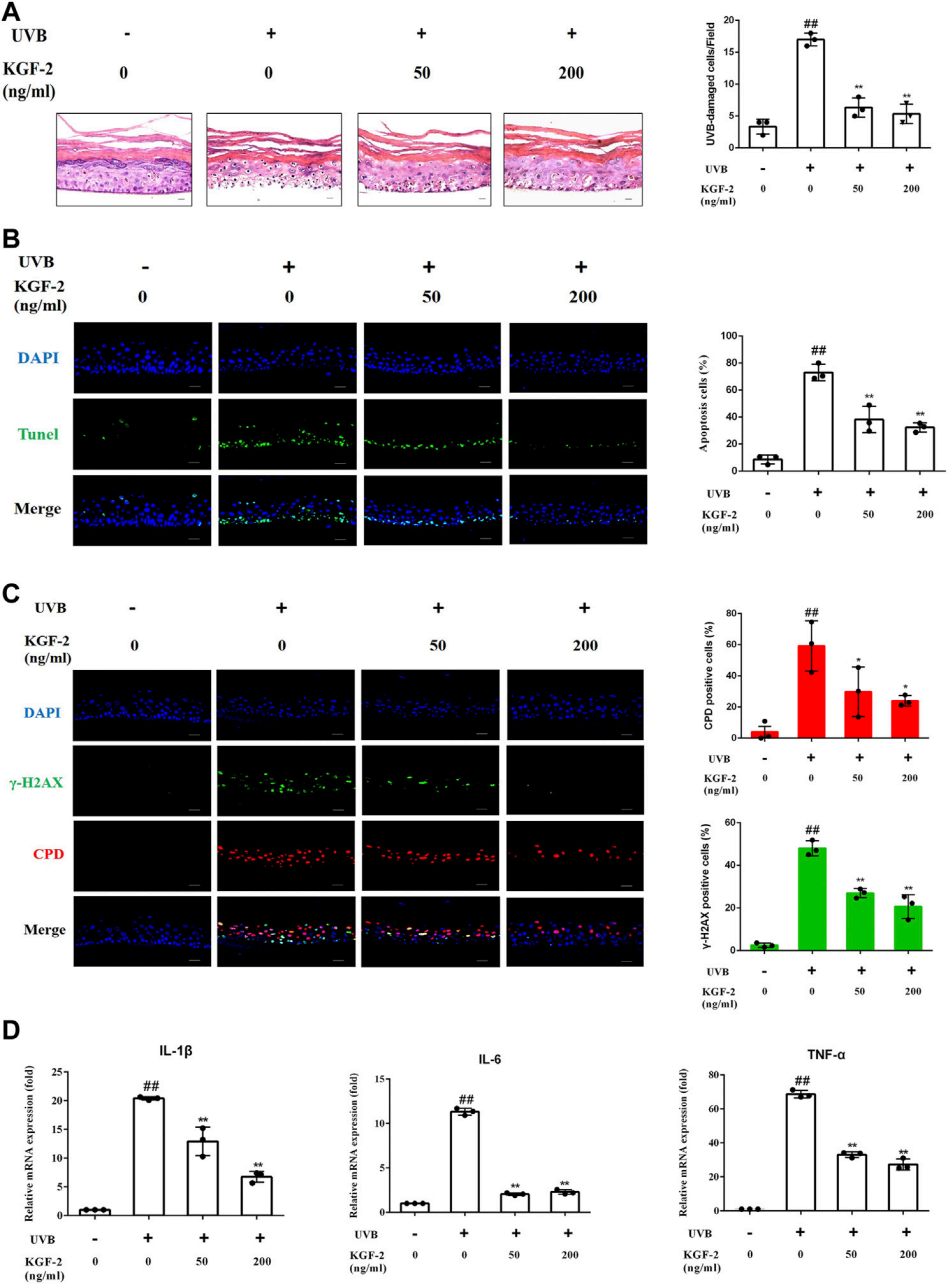
Effect of KGF-2 on UVB-irradiated HEE. HEE was treated with or without KGF-2 (0, 50, 200 ng/ml) for 4 h, and then irradiated with 200 mJ/cm^2^ dose of UVB. 24 h later, the HEE was collected and subjected to a series of analyses. **(A)** Specimens of HEE stained with HE, the plot compares the UVB-damaged cell counts taken from three independent HEE cultures. UVB only *vs.* untreated control group, *p* < 0.0001; KGF-2 (50 ng/ml) *vs.* UVB only group, *p* < 0.0001; KGF-2 (200 ng/ml) *vs.* UVB only group, *p* < 0.0001. **(B)** Representative image of immunofluorescence staining of apoptotic keratinocytes; the plot compares the apoptotic cell number within HEEs from three different treatments. UVB only *vs.* untreated control group, *p* < 0.0001; KGF-2 (50 ng/ml) *vs.* UVB only group, *p* = 0.0004; KGF-2 (200 ng/ml) *vs.* UVB only group, *p* = 0.0001 **(C)** Representative immunofluorescence images of HEEs stained with anti-CPD and anti-γ-H2AX antibodies, the first plot compares the formation of CPD of HEEs from three different treatments, UVB only *vs.* untreated control group, *p* = 0.0012; KGF-2 (50 ng/ml) *vs.* UVB only group, *p* = 0.0393; KGF-2 (200 ng/ml) *vs.* UVB only group, *p* = 0.0166. The second plot compares the formation of γ-H2AX of HEEs from three different treatments, UVB only *vs.* untreated control group, *p* < 0.0001; KGF-2 (50 ng/ml) *vs.* UVB only group, *p* = 0.0002; KGF-2 (200 ng/ml) *vs.* UVB only group, *p* < 0.0001. **(D)** qRT-PCR analysis of IL-1β,IL-6, and TNF-α mRNA levels in HEEs subjected to different treatments. For IL-1β, UVB only *vs.* untreated control group, *p* < 0.0001; KGF-2 (50 ng/ml) *vs.* UVB only group, *p* = 0.0004; KGF-2 (200 ng/ml) *vs.* UVB only group, *p* < 0.0001. For IL-6, UVB only *vs.* untreated control group, *p* < 0.0001; KGF-2 (50 ng/ml) *vs.* UVB only group, *p* < 0.0001; KGF-2 (200 ng/ml) *vs.* UVB only group, *p* < 0.0001. For TNF-α, UVB only *vs.* untreated control group, *p* < 0.0001; KGF-2 (50 ng/ml) *vs.* UVB only group, *p* < 0.0001; KGF-2 (200 ng/ml) *vs.* UVB only group, *p* < 0.0001. All graphical data are the means ± SD from three independent experiments, “#” and “##” indicate significantly different from the untreated control group at the *p* < 0.05 and *p* < 0.01 levels, respectively, “*” and “**” indicate significantly different from the UVB only control group at the *p* < 0.05 and *p* < 0.01 levels, respectively.

HEE irradiated with 200 mJ/cm^2^ dose of UVB followed by 24 h of culturing revealed a significant increase in the number of UVB-damaged keratinocyte (as shown by HE staining) compared with control (non-irradiated) HEE, but the number of UVB-damaged keratinocyte was reduced when HEE was pretreated with KFG-2 before UVB irradiation (Figure 1A). Consistent with the result obtained from HE staining, the result obtained from TUNEL assay also showed that KGF-2 could prevent the keratinocytes from undergoing apoptosis in the UVB-irradiated HEE (Figure 1B). UVB-induced DNA damage is crucial to the initiation of apoptosis*.* In general, DNA damage can be detected by looking at the formation of CPD and γ-H2AX within the HEE cells. DNA damage in the UVB-irradiated HEE was detected by the formation of CPD and γ-H2AX using immunofluorescence staining. No foci of CPD and γ-H2AX were observed within the cell nuclei of the control HEE, whereas many CPD and γ-H2AX foci were detected in the cell nuclei of UVB-irradiated HEE. The number of CPD and γ-H2AX foci in the cell nuclei was significantly reduced when the HEE was treated with KGF-2 before UVB irradiation (Figure 1C). UVB-irradiated keratinocytes are known to release various proinflammatory cytokines such as IL-1β, IL-6, and TNF-α (Kim et al., 2013). The mRNA levels of IL-1β,IL-6, and TNF-α were notably increased in UVB-irradiated HEE (Figure 1D). However, pretreatment with KGF-2 led to a reduction in the mRNA levels of all these proinflammatory cytokines. These results suggested that KGF-2 could prevent or reduce the extent of DNA damage and formation of associated cytokines in UVB-irradiated HEE, demonstrating the protective effect of KGF-2 against UVB-induced damage on the skin.

### Keratinocyte Growth Factor-2 Attenuates Apoptosis, Reactive Oxygen Species Production, and DNA Damage Caused by Ultraviolet B Exposure in Keratinocytes

**FIGURE 2:**
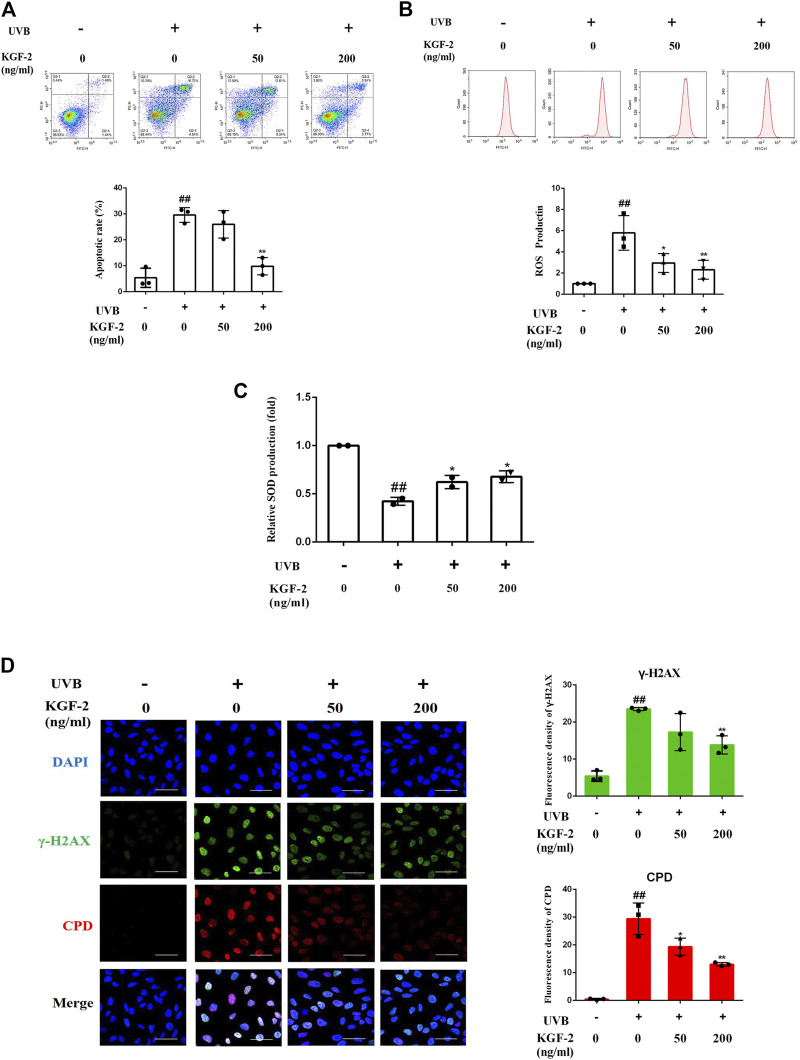
Effects of KGF-2 on the apoptosis, ROS, and SOD production as well as DNA damage of UVB-irradiated HaCaT cells. HaCaT cells were incubated with KGF-2 (0, 50, 200 ng/ml) for 4 h, then exposed to UVB (200 mJ/cm^2^) and further incubation for the indicated times. **(A)** Flow cytometry analysis of apoptotic HaCaT cells stained with Annexin V/PI after 6-h incubation following UVB irradiation, the plot compares the apoptotic rate from three different treatments. UVB only *vs.* untreated control group, *p* = 0.0002; KGF-2 (50 ng/ml) *vs.* UVB only group, *p* = 0.5635; KGF-2 (200 ng/ml) *vs.* UVB only group, *p* = 0.0007. **(B)** Intracellular ROS levels as measured by flow cytometry using the oxidant-sensitive probe DCFH-DA after 1-h incubation following UVB irradiation, the plot compares the relative ROS production from three different treatments. UVB only *vs.* untreated control group, *p* = 0.0012; KGF-2 (50 ng/ml) *vs.* UVB only group, *p* = 0.0242; KGF-2 (200 ng/ml) *vs.* UVB only group, *p* = 0.0084. **(C)** The SOD levels were measured after 6-h incubation following UVB irradiation. UVB only *vs.* untreated control group, *p* = 0.0008**;** KGF-2 (50 ng/ml) *vs.* UVB only group, *p* = 0.0385; KGF-2 (200 ng/ml) *vs.* UVB only group, *p* = 0.0168. **(D)** Representative image of immunofluorescence staining of CPD and γ-H2AX were obtained after incubating for 1-h following UVB irradiation, the plot compares the formation of CPD and γ-H2AX from three different treatments. For γ-H2AX, UVB only *vs.* untreated control group, *p* = 0.0002; KGF-2 (50 ng/ml) *vs.* UVB only group, *p* = 0.0703; KGF-2 (200 ng/ml) *vs.* UVB only group, *p* = 0.0085. For CPD, UVB only *vs.* untreated control group, *p* < 0.0001; KGF-2 (50 ng/ml) *vs.* UVB only group, *p* = 0.0132; KGF-2 (200 ng/ml) *vs.* UVB only group, *p* = 0.0007. All graphical data are the means ± SD from three independent experiments, “#” and “##” indicate significantly different from the untreated control group at the *p* < 0.05 and *p* < 0.01 levels, respectively, “*” and “**” indicate significantly different from the UVB only control group at the *p* < 0.05 and *p* < 0.01 levels, respectively.

At the cellular level, the exposure of keratinocytes to excessive UVB radiation can cause irreversible DNA damages and induce ROS production, which will eventually lead to apoptosis (Wang et al., 1999; Afaq et al., 2007). After UVB irradiation, the number of apoptotic cells was significantly elevated compared with the control (non-irradiated) sample, but the apoptotic rate was significantly repressed when the cells were treated with KGF-2 before UVB irradiation, indicating that KGF-2 could protect HaCaT cells against UVB-induced apoptosis (Figure 2A). UVB irradiation also stimulated the high production of intracellular ROS compared with the control cells. Treatment with 50 or 200 ng/ml KGF-2 before UVB irradiation significantly reduced ROS production (Figure 2B). Interestingly, KGF-2 pretreatment reversed the loss of key antioxidant enzymes, e.g., superoxide dismutase (SOD), in UVB-injured HaCaT cells (Figure 2C). The level of CPD and γ-H2AX in UVB-irradiated HaCaT cells increased significantly compared with the control cells. Pretreatment of HaCaT cells with KGF-2 significantly decreased the formation of CPD and γ-H2AX (Figure 2D). These results indicated that KGF-2 could protect keratinocytes from UVB-induced cell damage.

### Keratinocyte Growth Factor-2 Ameliorates Mitochondrial Dysfunction Caused by Ultraviolet B Irradiation

**FIGURE 3:**
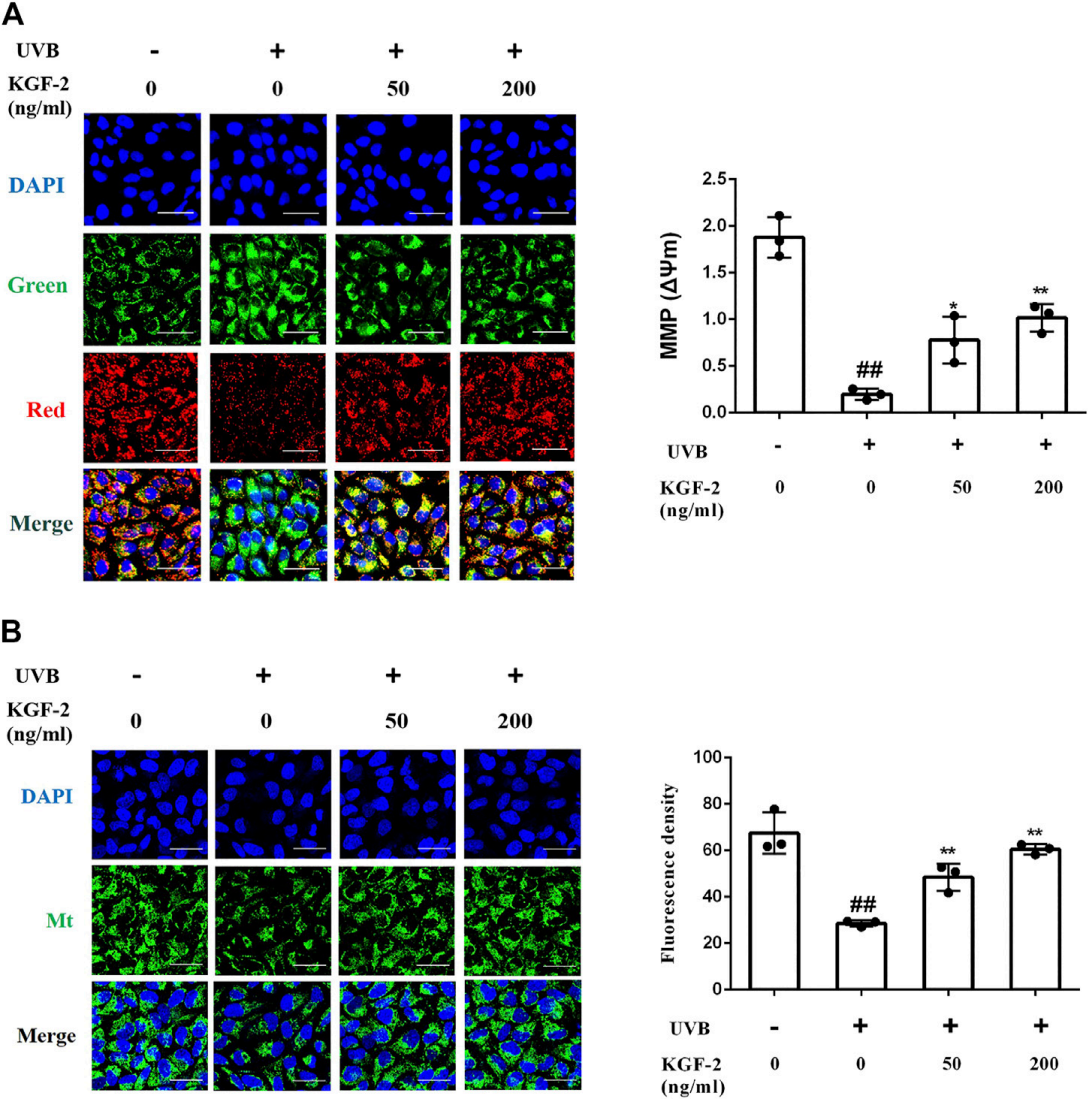
Effect of KGF-2 on the mitochondrial dysfunction induced by of UVB irradiation. HaCaT cells were incubated with KGF-2 (0, 50, 200 ng/ml) for 4 h, then exposed to UVB (200 mJ/cm^2^) and a further 1-h incubation. **(A)** Representative image of mitochondrial membrane potential (MMP) in HaCaT cells, the plot compares the MMP of HaCaT cells from three different treatments. UVB only *vs.* untreated control group, *p* < 0.0001; KGF-2 (50 ng/ml) *vs.* UVB only group, *p* = 0.0125; KGF-2 (200 ng/ml) *vs.* UVB only group, *p* = 0.0016. **(B)** Representative image of mitochondrial mass, the plot compares the mitochondrial mass of HaCaT cells from three different treatments. UVB only *vs.* untreated control group, *p* < 0.0001; KGF-2 (50 ng/ml) *vs.* UVB only group, *p* = 0.0057; KGF-2 (200 ng/ml) *vs.* UVB only group, *p* = 0.0003. All graphical data are the means ± SD from three independent experiments, “#” and “##” indicate significantly different from the untreated control group at the *p* < 0.05 and *p* < 0.01 levels, respectively, “*” and “**” indicate significantly different from the UVB only control group at the *p* < 0.05 and *p* < 0.01 levels, respectively.

UVB-induced ROS overproduction can result in mitochondrial dysfunction, a major cause of apoptosis. To evaluate the effect of KGF-2 on mitochondrial function, the mitochondrial membrane potential (MMP) and mitochondrial mass of HaCaT cells were measured following UVB irradiation. HaCaT cells irradiated with UVB displayed a loss of MMP while those that had been treated with KGF-2 before UVB irradiation showed an elevated level of MMP (Figure 3A)*.* In agreement with the result of MMP, KGF-2 also significantly attenuated the loss of mitochondrial mass caused by UVB irradiation (Figure 3B). Thus, KGF-2 could prevent mitochondrial dysfunction in HaCaT cells induced by UVB irradiation.

### Keratinocyte Growth Factor-2 Activates the Aryl Hydrocarbon Receptor/Nrf2 Signaling Pathway

**FIGURE 4:**
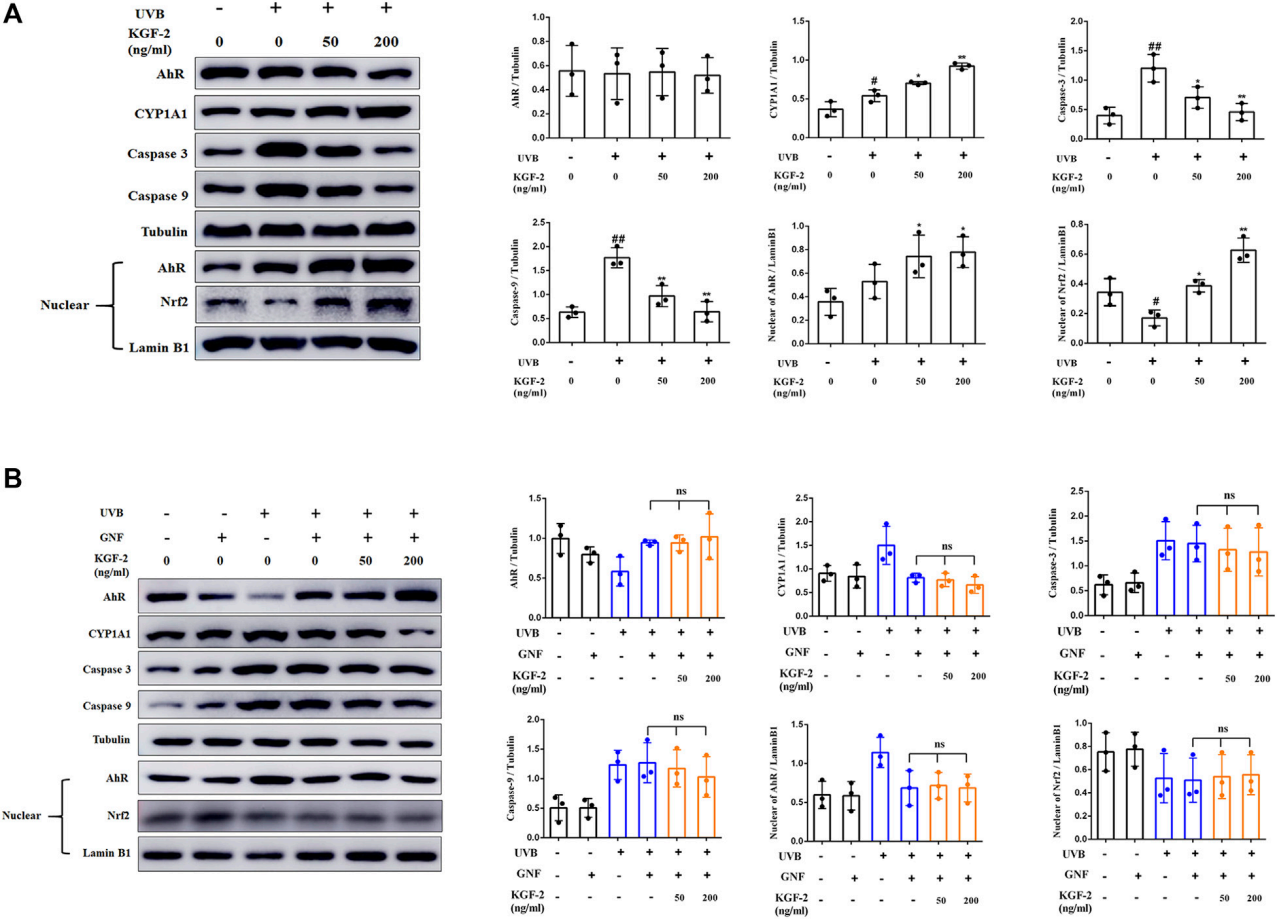
Effects of KGF-2 on the AhR/Nrf2 signaling pathway in keratinocytes following UVB irradiation. **(A)** HaCaT cells were treated with or without KGF-2 for 4 h followed by exposure to UVB (200 mJ/cm^2^) and a further 30-min incubation. The cells were then subjected to western blot analysis to measure the levels of AhR, CYP1A1, caspase 3, caspase 9 in cytoplasm and the levels of AhR, and Nrf2 in nucleus, the plot compares the protein level of HaCaT cells from three different experiments. AhR: UVB only *vs.* untreated control group, *p* = 0.9976; KGF-2 (50 ng/ml) *vs.* UVB only group, *p* = 0.9995; KGF-2 (200 ng/ml) *vs.* UVB only group, *p* = 0.9995. CYP1A1: UVB only *vs.* untreated control group, *p* = 0.0288; KGF-2 (50 ng/ml) *vs.* UVB only group, *p* = 0.0378; KGF-2 (200 ng/ml) *vs.* UVB only group, *p* = 0.0003. Caspase 3: UVB only *vs.* untreated control group, *p* = 0.0015; KGF-2 (50 ng/ml) *vs.* UVB only group, *p* = 0.0237; KGF-2 (200 ng/ml) *vs.* UVB only group, *p* = 0.0025. Caspase 9: UVB only *vs.* untreated control group, *p* = 0.0003; KGF-2 (50 ng/ml) *vs.* UVB only group, *p* = 0.0025; KGF-2 (200 ng/ml) *vs.* UVB only group, *p* = 0.0003. AhR in nucleus: UVB only *vs.* untreated control group, *p* = 0.3802; KGF-2 (50 ng/ml) *vs.* UVB only group, *p* = 0.2422; KGF-2 (200 ng/ml) *vs.* UVB only group, *p* = 0.1558. Nrf2 in nucleus: UVB only *vs.* untreated control group, *p* = 0.0390; KGF-2 (50 ng/ml) *vs.* UVB only group, *p* = 0.0131; KGF-2 (200 ng/ml) *vs.* UVB only group, *p* = 0.0001. **(B)** HaCaT cells were pretreated with or without GNF351 for 12 h followed by identical treatment as western blot analysis as in A. AhR: KGF-2 (50 ng/ml) *vs.* UVB + GNF group, *p* = 0.9999; KGF-2 (200 ng/ml) *vs.* UVB + GNF group, *p* = 0.9746. CYP1A1: KGF-2 (50 ng/ml) *vs.* UVB + GNF group, *p* = 0.9987; KGF-2 (200 ng/ml) *vs.* UVB + GNF group, *p* = 0.9746. Caspase 3: KGF-2 (50 ng/ml) *vs.* UVB + GNF group, *p* = 0.9898; KGF-2 (200 ng/ml) *vs.* UVB + GNF group, *p* = 0.9650. Caspase 9: KGF-2 (50 ng/ml) *vs.* UVB + GNF group, *p* = 0.9904; KGF-2 (200 ng/ml) *vs.* UVB + GNF group, *p* = 0.7419. AhR in nucleus: KGF-2 (50 ng/ml) *vs.* UVB + GNF group, *p* = 0.9997; KGF-2 (200 ng/ml) *vs.* UVB + GNF group, *p* = 0.9999. Nrf2 in nucleus: KGF-2 (50 ng/ml) *vs.* UVB + GNF group, *p* = 0.9997; KGF-2 (200 ng/ml) *vs.* UVB + GNF group, *p* = 0.9971. All graphical data are the means ± SD from three independent experiments, “#” and “##” indicate significantly different from the untreated control group at the *p* < 0.05 and *p* < 0.01 levels, respectively, “*” and “**” indicate significantly different from the UVB only control group at the *p* < 0.05 and *p* < 0.01 levels, respectively.

Apoptosis-related caspase-3 and caspase-9 can be activated in UVB-irradiated keratinocytes (Kamarajan and Chao 2000; Itoh and Horio 2001). Pretreatment of HaCaT cells with KGF-2 resulted in a reduction of the caspase-3 and caspase-9 activation, indicating that KGF-2 could effectively inhibit UVB-induced apoptosis (Figure 4A). It has been reported that the aryl hydrocarbon receptor (AhR) is a crucial mediator of the UVB stress response in human keratinocytes following UVB exposure (Fritsche et al., 2007). In unstressed cells, ligand-free AhRs are maintained in an inactive complex in the cytosol. In the epidermal compartment, UVB is absorbed by the aromatic amino acid L-tryptophan, leading to the formation of 6-formylindolo (3,2-b)carbazole (FICZ), which binds AhR with high affinity and induces pp60src to dissociate from the complex. The AhR is then translocated from the cytoplasm to the nucleus where it heterodimerizes with ARNT and induces the expression of monooxygenase cytochrome P450 (CYP1A1) (Villard et al., 2002; Agostinis et al., 2007; Fritsche et al., 2007; Diani-Moore et al., 2011). HaCaT cells that were irradiated with UVB exhibited a significant increase in the level of AhR in the nuclei compared with the untreated cells. However, when the cells were pretreated with KGF-2, the translocation of AhR into the nucleus was promoted (Figure 4A). Furthermore, these cells also displayed a marked increase in CYP1A1 expression compared with those irradiated with UVB without KGF-2 pretreatment (Figure 4A). In inactive state, Nrf2 is anchored to Kelch-like ECH associated protein 1 (Keap1) in the cytoplasm. Once activated, Nrf2 departs from Keap1, leading to its stabilization, accumulation, and translocation to nuclei (Itoh et al., 2004; Li and Kong 2009; Suzuki and Yamamoto 2015). Our data showed that KGF-2 could promote the translocation of Nrf2 to the nucleus, indicating that KGF-2 could activate the Nrf2 signaling pathway. In order to clarify whether KGF-2 could activate Nrf2 through activating AhR, HaCaT cells were pretreated with GNF351 (AhR antagonist) for 12 h prior to treatment with KGF-2 in order to block the translocation of AhR into the nucleus. After UVB irradiation, the expression of AhR in the nucleus of these cells was significantly inhibited compared with the cells that were not treated with GNF351, indicating that UVB induced AhR activation was effectively blocked by GNF351. The result also demonstrated when the activation of AhR was blocked, KGF-2 could not elevate the expression of CYP1A1 and inhibit the expression of caspase-3 and caspase-9 after UVB irradiation. More importantly, KGF-2 could not trigger Nrf2 activation once the activation of AhR was blocked, demonstrating that KGF-2 could stimulate the activation of Nrf2 *via* activating the AhR pathways (Figure 4B).

### Keratinocyte Growth Factor-2 Protects HaCaT Cells From Ultraviolet B-Induced Damage Through Activating Aryl Hydrocarbon Receptor/Nrf2 Signaling Pathway

**FIGURE 5:**
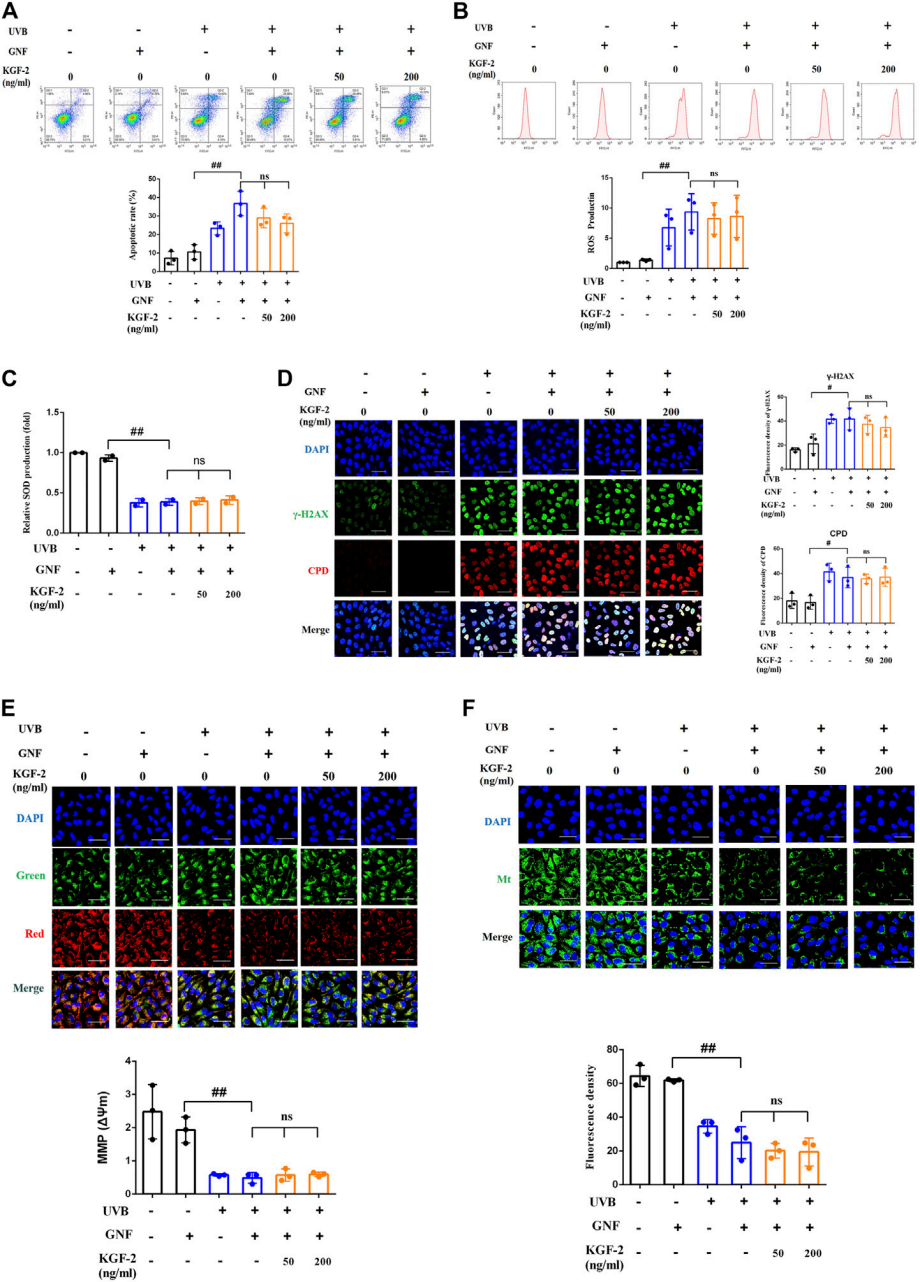
HaCaT cells were pretreated with or without GNF351 for 12 h prior and then treated with KGF-2. After that, the cells were treated with or without KGF-2 for 4 h, and then exposed to UVB (200 mJ/cm^2^). The protective effect of KGF-2 on the UVB-induced cell damage was then determined. **(A)** Flow cytometric analysis of apoptotic HaCaT cells, the plot compares the apoptotic rates from three different treatments. UVB + GNF *vs.* GNF group, *p* = 0.0001; KGF-2 (50 ng/ml) *vs.* UVB + GNF group, *p* = 0.2314; KGF-2 (200 ng/ml) *vs.* UVB + GNF group, *p* = 0.0675. **(B)** Representative image of intracellular ROS levels; The plot compares the relative ROS production from three different treatments. UVB + GNF *vs.* GNF group, *p* = 0.0083; KGF-2 (50 ng/ml) *vs.* UVB + GNF group, *p* = 0.9729; KGF-2 (200 ng/ml) *vs.* UVB + GNF group, *p* = 0.9952. **(C)** SOD levels from different treatments. UVB + GNF *vs.* GNF group, *p* < 0.0001; KGF-2 (50 ng/ml) *vs.* UVB + GNF group, *p* = 0.9988; KGF-2 (200 ng/ml) *vs.* UVB + GNF group, *p* = 0.9588. **(D)** Representative image of immunofluorescence staining of CPD and γ-H2AX, the plot compares the formation of CPD and γ-H2AX from three different treatments. γ-H2AX: UVB + GNF *vs.* GNF group, *p* = 0.0128; KGF-2 (50 ng/ml) *vs.* UVB + GNF group, *p* = 0.8935; KGF-2 (200 ng/ml) *vs.* UVB + GNF group, *p* = 0.6063. CPD: UVB + GNF *vs.* GNF group, *p* = 0.0155; KGF-2 (50 ng/ml) *vs.* UVB + GNF group, *p* = 0.9997; KGF-2 (200 ng/ml) *vs.* UVB + GNF group, *p* = 0.9999. **(E)** Representative image of mitochondrial membrane potential (MMP) in HaCaT cells, the plot compares the MMP of HaCaT cells from three different treatments. UVB + GNF *vs.* GNF group, *p* = 0.0002; KGF-2 (50 ng/ml) *vs.* UVB + GNF group, *p* = 0.99855; KGF-2 (200 ng/ml) *vs.* UVB + GNF group, *p* = 0.9961. **(F)** Representative image of mitochondrial mass, the plot compares the mitochondrial mass of HaCaT cells from three different treatments. UVB + GNF *vs.* GNF group, *p* < 0.0001; KGF-2 (50 ng/ml) *vs.* UVB + GNF group, *p* = 0.8163; KGF-2 (200 ng/ml) *vs.* UVB + GNF group, *p* = 0.7211. All graphical data are the means ± SD from three independent experiments, “#” and “##” indicate significantly different from the GNF only control group at the *p* < 0.05 and *p* < 0.01 levels, respectively, “*” and “**” indicate significantly different from the UVB + GNF group at the *p* < 0.05 and *p* < 0.01 levels, respectively.

In order to clarify whether KGF-2 could protect the keratinocytes from UVB-induced damage through activating AhR/Nrf2 signaling pathway, HaCaT cells were pretreated with or without GNF351 for 12 h prior to KGF-2 treatment. The extent of UVB-induced keratinocytes apoptosis was then determined by flow cytometry. The result showed that KGF-2 could not inhibit the apoptotic rate induced by UVB irradiation when the cells were pretreated with GNF351, suggesting that blocking the activation of AhR prevented the KGF-2 from conferring its protective effect against UVB-induced apoptosis (Figure 5A). Pretreatment of HaCaT cells with GNF351 followed by KGF-2 treatment also led to the lack of reduction of ROS production following UVB irradiation compared with no GNF351 pretreatment, indicating that the ability of KGF-2 to reduce the production of ROS induced by UVB irradiation was abolished once the activation of AhR was blocked (Figure 5B). Under this condition, KGF-2 could not prevent the reduction of SOD and formation of CPD and γ-H2AX and protect the mitochondria against the loss function induced by UVB irradiation (Figures 5C–F), suggesting that KGF-2 might protect the HaCaT cells from UVB-induced damage *via* activating AhR/Nrf2 pathway.

## Discussion

UV radiation is the most prominent and ubiquitous physical stressor in our environment and the gradual depletion of the stratospheric ozone over the past many decades has resulted in more intensive UV radiation reaching the Earth’s surface. This has translated into more UV-induced damage to individuals without proper protection while exposing their skins to direct sunlight, especially in summertime. Such UV-induced damages include erythema, oedema, heat, burn, and pruritus.

KGF might provide efficient photoprotection of the human skin against UV radiation because it could markedly reduce cell death and the intracellular level of ROS, while preventing the loss of catalase activity in the epidermis after UVB irradiation (Braun et al., 2006; Kovacs et al., 2009). KGF-2 is a typical paracrine growth factor, and it has been shown to possess anti-inflammatory and antioxidation activities, increase cell proliferation as well as reducing apoptosis through initiating the activation of intracellular signaling cascades. These signaling cascades include the extracellular signal-regulated kinase (ERK) 1/2 signaling pathway, phosphatidylinositol-3 kinase/protein kinase B (PI3K/Akt) signaling pathway, and Nrf2 signaling pathway (Upadhyay et al., 2005; Shi et al., 2018; Dong et al., 2019; Tan et al., 2020). Previous studies have focused on the role of KGF-2 in embryonic development, wound healing, and tissue regeneration. However, the effect of KGF-2 on UVB-induced skin damage was not clear. We have shown that KGF-2 could ameliorate UVB-induced skin damage through inhibiting oxidative stress, DNA damage, apoptosis, and mitochondrial dysfunction.

The ability of KGF-2 to ameliorate the extent of UVB-induced epidermis damage was demonstrated using human epidermal equivalent (HEE) as a skin substitute. HEE was used instead of animals because it could faithfully mimic the *in vivo* epidermis (Smits et al., 2017; Yoshida et al., 2012). A number of parameters were monitored to demonstrate the level of damage sustained by HEE following UVB irradiation. HEE irradiated with UVB clearly exhibited an increase in the number of UVB-damaged cells accompanied by DNA damage and inflammation. However, treatment of the HEE with KGF-2 before UVB irradiation resulted in marked protection against the UVB radiation-induced damage, as seen by the reduced number of UVB-damaged cells, lesser DNA damage as well as lower inflammation response. UV radiation can cause inflammation, aging, and cell damage (Kim et al., 2019). If the dose of UVB sustained by the keratinocytes exceeds a threshold damage response, apoptosis will be activated, leading to the death of these cells as a mean to prevent the accumulation of potentially mutagenic keratinocytes within the skin (Van Laethem et al., 2005; Nakanishi et al., 2009; Deshmukh et al., 2017). DNA is the most abundant chromophore present in the epidermis, UVB is directly absorbed by DNA which causes molecular rearrangements forming the photoproducts such as CPD and 6–4 photoproducts (D'Orazio et al., 2013; Chen et al., 2014). CPDs are the main DNA damage lesions responsible for cell death following UV exposure (Kciuk and Marciniak 2020). Increased formation of CPD can lead to an increase in the phosphorylation of the histone protein H2AX at Ser139. The phosphorylated H2AX, termed γ-H2AX, is not only an important biomarker of DNA damage, but is also an important marker of apoptosis (Zhao et al., 2014; Huang et al., 2003). KGF-2 also decreased the formation of CPD and γ-H2AX in HEE and HaCaT cell (Figures 1C, 2D), indicating that KGF-2 could protect the epidermis from UVB induced DNA damage. One of the most obvious acute effects of UV on the skin is the induction of inflammation. Several studies have shown that UVB stimulates release of IL-1β, IL-6, and TNF-α in epidermis, which cause pain and redness (Rabe et al., 2006; Oh et al., 2014; Prasad and Katiyar 2017). Our data explicitly showed that KGF-2 was able to inhibit the production of proinflammatory cytokines (IL-1β, IL-6, and TNF-α) in UVB-irradiated HEEs, consistent with our expectation (Figure 1D).

Changes in the skin following UV exposure are associated with changes at the cellular level. HaCaT, a spontaneously immortalized human keratinocyte cell line, possesses similar biological characteristics as those found in primary keratinocytes, making them suitable for studying UVB-induced epidermal damage *in vitro*. Several experiments were performed to determine whether KGF-2 could reduce UVB-induced damage in HaCaT cell. HaCaT cells irradiated with UVB clearly exhibited increased apoptotic rate and DNA damage, ROS overproduction and loss of SOD as well as the presence of mitochondrial dysfunction. The concurrent increase in ROS production and inhibition of antioxidant defense mechanism could result in mitochondrial dysfunctions (Yoon et al., 2010; Hwang et al., 2014; Kudryavtseva et al., 2016). These effects appeared to be suppressed by KGF-2, clearly demonstrating a protective role for KGF-2 in the reduction of oxidative stress, DNA damage, apoptosis, and mitochondrial dysfunction in HaCaT cells, providing a further evidence that KGF-2 may be crucial for an efficient skin photoprotection.

UVB is absorbed by the aromatic amino acid tryptophan present in the cells*,* leading to the formation of FICZ that binds AhR with high affinity to regulate the expression of several genes with toxic or protective effects (Fritsche et al., 2007; Diani-Moore et al., 2011), (Nebert 2017; Rothhammer and Quintana 2019). UVB irradiation could trigger the translocation of AhR from the cytosol to the nucleus in HaCaT cells, suggesting its activation by UVB. Surprisingly, KGF-2 pretreatment followed by UVB-irradiation resulted in further increase in nuclear AhR, indicating that the activation of AhR was enhanced by KGF-2 (Figure 4A). AhR mediates the cutaneous stress response toward a variety of environmental noxae and therefore it has attracted great interest in the field of modern preventive medicine (Haarmann-Stemmann et al., 2012). Several studies have demonstrated that AhR can contribute to the pathogenesis of some skin diseases. Indeed, the activation of AhR might be advantageous to inflammation, skin barrier function, and oxidative stress. AhR has been considered as a possible therapeutic target for the treatment of skin diseases such as atopic dermatitis, psoriasis, acne, and vitiligo (Napolitano and Patruno 2018). The antioxidant response of AhR is thought to be mediated through the activation of Nrf2, since the transcription of Nrf2 is directly regulated by AhR (Miao et al., 2005). In the inactive state, Nrf2 is anchored to Keap1 in the cytoplasm. Once activated, Nrf2 departs from Keap1, leading to its stabilization, accumulation and translocation to nuclei, where it will bind to the antioxidant responsive element (ARE) on a number of antioxidant genes and regulate their expression (Itoh et al., 2004; Li and Kong 2009; Suzuki and Yamamoto 2015). The ability of KGF-2 to promote the translocation of Nrf2 to the nucleus and increase the expression of CYP1A1 suggested that KGF-2 could activate Nrf2 (Figure 4A). Previous studies have shown that Nrf2 plays a beneficial role in protecting skin against UVB-induced inflammation, oxidative damage, cellular dysfunction, and sunburn reaction in the skin (Saw et al., 2011; Ikehata and Yamamoto 2018). Nrf2 activation plays a critical role in the photoprotection of skin. Nrf2 deficiency exacerbates UVB-induced skin damage such as inflammation, DNA damage, and extracellular matrix damage, while the activation of Nrf2 can protect against UV-triggered skin carcinogenesis (Saw et al., 2014; Knatko et al., 2015), (Li et al., 2017; Rojo de la Vega et al., 2018; Gęgotek et al., 2018; Chaiprasongsuk et al., 2019). The AhR antagonist GNF351 can interact directly with the ligand-binding pocket and compete with a well-characterized photoaffinity AhR ligand for binding with AhR that ultimately results in the blocking of AhR nuclear translocation (van den Bogaard et al., 2015). The activation of Nrf2 triggered by KGF-2 was substantially suppressed by the AhR antagonist GNF351, indicating that KGF-2 could activate Nrf2 to exert its antioxidant effect through activating AhR after UVB irradiation. Furthermore, the ability of KGF-2 to reduce cell apoptosis and ROS production, elevate the SOD production, ameliorate DNA damage, and mitochondrial dysfunctions was completely abolished once the activation of AhR was blocked. These results demonstrated for the first time that KGF-2 may stimulate the activation of Nrf2 *via* activating the AhR pathway*,* thereby protecting the keratinocytes from UVB-induced cell damage. However, the precise mechanism of how KGF-2 might activate AhR is a subject of further investigation.

## Conclusion

KGF-2 could successfully prevent UVB-induced HEE damage and significantly inhibit UVB-induced DNA damage and inflammatory response. In addition, KGF-2 could alleviate UVB-induced keratinocytes apoptosis, ROS production, DNA damage, and mitochondrial dysfunction and up-regulate SOD production in HaCaT cells. The protective mechanism of KGF-2 on UVB-induced skin damage might involve AhR-activated Nrf2 signaling. Our research could serve as a theoretical and experimental basis for the development and application of KGF-2 in protecting human skin against UVB irradiation.

## Data Availability

The raw data supporting the conclusions of this article will be made available by the authors, without undue reservation, to any qualified researcher.
